# Clinical significance of antibodies to Ro52/TRIM21 in systemic sclerosis

**DOI:** 10.1186/ar3763

**Published:** 2012-03-06

**Authors:** Marie Hudson, Janet Pope, Michael Mahler, Solène Tatibouet, Russell Steele, Murray Baron, Marvin J Fritzler

**Affiliations:** 1 Division of Rheumatology, Jewish General Hospital, 3755 Cote Ste Catherine, Montréal H3T 1E3, Quebec, Canada; 2 Lady Davis Research Institute, Jewish General Hospital, 3755 Cote Ste Catherine, Montréal H3T 1E3, Quebec, Canada; 3 Faculty of Medicine, Division of Rheumatology, University of Western Ontario, St. Joseph's Health Centre, 268 Grosvenor Street, London N6A 4V2, Ontario, Canada; 4 INOVA Diagnostics, Inc, 9900 Old Grove Rd, San Diego, CA 32131-1638, USA; 5 Faculty of Medicine, University of Calgary, 3330 Hospital Drive, Calgary T2N 4N1, Alberta, Canada

## Abstract

**Introduction:**

Autoantibodies to Ro52 recently identified as TRIM21 are among the most common autoantibodies in systemic autoimmune rheumatic diseases, but their clinical association remains poorly understood. We undertook this study to determine the clinical and serologic associations of anti-Ro52/TRIM21 antibodies in patients with systemic sclerosis (SSc).

**Methods:**

Detailed clinical data and sera from 963 patients with SSc enrolled in a multicenter cohort study were collected and entered into a central database. Antibodies to Ro52/TRIM21 and other autoantibodies were detected with an addressable laser-bead immunoassay and different enzyme-linked immunosorbent assay (ELISA) systems. Associations between anti-Ro52/TRIM21 antibodies and clinical and other serologic manifestations of SSc were investigated.

**Results:**

Anti-Ro52/TRIM21 antibodies were present in 20% of SSc patients and overlapped with other main SSc-related antibodies, including anti-centromere (by immunofluorescence and centromere protein (CENP)-A and CENP-B ELISA), anti-topoisomerase I, anti-RNA polymerase III, and anti-Pm/Scl antibodies. Anti-Ro52/TRIM21 antibodies were strongly associated with interstitial lung disease (odds ratio (OR), 1.53; 95% confidence interval (CI), 1.11 to 2.12; *P *= 0.0091) and overlap syndrome (OR, 2.06; 95% CI, 1.01 to 4.19; *P *= 0.0059).

**Conclusions:**

Anti-Ro52/TRIM21 antibodies were the second most common autoantibodies in this SSc cohort. In SSc, anti-Ro52/TRIM21 antibodies may be a marker of interstitial lung disease and overlap syndrome.

## Introduction

Systemic sclerosis (SSc; scleroderma) is a disorder characterized by fibrosis of the skin and visceral organs. The pathogenesis of this disease is complex and remains incompletely understood. Nevertheless, autoantibodies represent a serologic hallmark of the disease and have proven value as diagnostic and prognostic biomarkers. Indeed, up to 95% of SSc patients [1] have circulating autoantibodies directed against one or more autoantigens, including topoisomerase I (formerly called Scl-70), centromere proteins (CENPs), RNA polymerase III, and the PM/Scl complex, also known as the human exosome [2]. In SSc, the major disease-related autoantibodies tend to be mutually exclusive [3], suggesting unique pathways for the induction of the B-cell response in this condition. Evidence supporting this concept is extensive, given data indicating that each autoantibody is associated with specific demographic, clinical, genetic, and prognostic features [4,5]. In addition, a growing knowledge of the role of SSc autoantibodies in the pathogenesis of the disease is helping to gain a better understanding of potential novel modes of therapy [6-9].

Two main types of SS-A/Ro antibodies have been described in SSc. One is directed at a 60-kDa protein known as SS-A/Ro60, which is part of a small cytoplasmic ribonucleoprotein (scRNP) multiprotein complex. Another, which often coexists with the former SS-A/Ro60 antibodies, is directed against a 52-kDa (Ro52) protein that is not normally part of the scRNP complex but is an E3 ubiquity ligase and a member of the tripartite motif (TRIM) family of proteins known as TRIM21 [10]; hence, the preferred terminology of Ro52/TRIM21 is used in this report. The association of Ro60 antibodies with autoimmune conditions is well established, particularly in systemic lupus erythematosus (SLE), subacute cutaneous lupus, and Sjögren syndrome (SjS). Ro52/TRIM21 antibodies have also been reported in a wide variety of autoimmune diseases, although often overlapping with other autoantibodies. However, little is known of their clinical associations, and controversy still exists about whether they have an independent association with autoimmune diseases [11].

The Canadian Scleroderma Research Group (CSRG) is a pan-Canadian, multicenter group of researchers that has, since 2004, recruited more than 1,200 SSc patients. The exact prevalence of SSc in Canada remains unknown, but estimates range from 70 to 440 cases/million [12,13]. Thus, by using the most conservative numbers, the CSRG currently captures up to 8% of all Canadian SSc cases. Detailed demographic, clinical, and serologic data have been obtained on these patients and entered into a central database.

Ro52/TRIM21 antibodies have been identified in 20% of the CSRG cohort, making it the second most common autoantibody in this cohort of SSc patients (Table 1). Given its high prevalence and the paucity of data on its clinical significance, we undertook this study to determine whether Ro52/TRIM21 antibodies in SSc are associated with distinct disease manifestations in SSc.

**Table 1:** Frequency of selected autoantibodies in the CSRG cohort

	*n*	%	Numbers not tested
CENP-B	285	36%	162
Centromere by IIF	334	35%	2
CENP-A	275	34%	162
Ro52/TRIM21	194	20%	0
RNA polymerase III	144	19%	188
Topoisomerase I	150	16%	0
PM/Scl	53	7%	188
Ro60	53	6%	0
U1 RNP	59	6%	0
Chromatin	44	5%	0
Sm	39	4%	0
SS-B/La	26	3%	0
Jo-I	6	1%	0
Ribosomal P	14	1%	0

*N *= 963. CENP, centromere protein; IIF, indirect immunofluorescence; PM/Scl, polymyositis/scleroderma (exosome) autoantigen; RNP, ribonucleoprotein; Sm, Smith autoantigen; SS-B, Sjögren syndrome antigen B or La.

## Methods

### Design

This is a cross-sectional study of a cohort of SSc patients.

#### Study subjects

The study subjects consisted of those enrolled in the Canadian Scleroderma Research Group (CSRG) registry. Patients in this registry are recruited from the practices of rheumatologists across Canada. They must have a diagnosis of SSc confirmed by a rheumatologist, be 18 years of age or older, be fluent in English or French, and be likely to be compliant with study procedures and visits. The patients available for this study were those whose baseline visit was between September 2004 and February 2011.

Certain features of our cohort, including age, female distribution, and proportion of patients with diffuse disease, suggest that the patients included in the CSRG registry are similar to patients included in other large SSc cohorts that have been assembled both in the United States and abroad [14]. Moreover, the cohort includes a mix of patients covering the spectrum of disease severity. The participating rheumatologists in the CSRG include both academic and community rheumatologists, but all have a particular interest in SSc, and thus all are perceived as "experts." They thus recruit patients with more severe disease. Conversely, because the American College of Rheumatology classification criteria for SSc exclude many patients with limited cutaneous disease [15,16], the patients in the CSRG Registry do not have to meet those criteria to be included. Thus, participating rheumatologists also recruit patients with probably milder disease. Finally, the patients in the CSRG Registry are generally recruited as outpatients, and the mean disease duration is approximately 10 years. The cohort probably includes survivor patients with less aggressive disease, but who may have accumulated damage over time. In general, we believe that our patients are representative of the spectrum of SSc seen by the general rheumatology community in Canada.

#### Measurement of autoantibodies

Serum was collected from all patients recruited by the CSRG and sent to a central laboratory, Mitogen Advanced Diagnostics Laboratory, at the University of Calgary. Aliquots of sera were stored at -70°C until needed. Anti-Ro52/TRIM21 and other related autoantibodies (topoisomerase I, chromatin, Sm, U1-RNP, ribosomal P, Jo-1, SSA/Ro60, SSB-La) were assayed with an addressable laser-bead immunoassay (ALBIA) by using a commercially available kit (QUANTAPlex ENA 8; INOVA Diagnostics Inc., San Diego, CA, USA) in a Luminex 100 (Luminex Corp., Austin, TX, USA) platform, according to protocols previously described [17]. In addition, anti-CENP antibodies were assessed with indirect immunofluorescence on an HEp-2000 substrate (ImmunoConcepts Inc., Sacramento, CA, USA), and antibodies to RNA polymerase III were detected with ELISA (INOVA Diagnostics) [17], as were antibodies to PM/Scl (PM1 alpha; Dr. Fooke Laboratorien GmbH, Neuss, Germany) [18]. CENP-B ELISA (recombinant full-length CENP-B), and CENP-A ELISA (Dr. Fooke Laboratorien GmbH) were performed according to the manufacturer's AI-Line instructions for use, as previously described [19].

#### Study measures

Patients recruited into the Registry undergo an extensive medical evaluation with standardized reporting of history, physical evaluation, and laboratory investigations. Demographic information regarding age, sex, and ethnicity is collected by patient self-report. Disease duration determined from the onset of the first non-Raynaud disease manifestation is recorded by the study physician. Skin involvement is assessed by using the modified Rodnan skin score [20], a widely used clinical assessment in which the examining rheumatologist records the degree of skin thickening, ranging from 0 (no involvement) to 3 (severe thickening) in 17 areas (total score range, 0 to 51), and patients are classified into limited and diffuse cutaneous subsets, based on the definition of Leroy *et al. *[21]. Joint examinations are performed by a rheumatologist by using the simplified 28 swollen and tender joint count [22]. History of inflammatory myositis, thrombosis, scleroderma renal crisis, and overlap syndrome was recorded by the study physician. For the purposes of this study, overlap syndrome was defined as a patient with SSc and SLE, SjS, rheumatoid arthritis, polymyositis/dermatomyositis, and/or mixed connective tissue disease.

To assess gastrointestinal involvement, patients answered yes/no to a series of 14 questions concerning appetite loss, difficulty swallowing, regurgitation of acid, nocturnal choking, heartburn, early satiety, abdominal bloating, nausea and vomiting, constipation, diarrhea, need for antibiotics for diarrhea, greasy stools, fecal incontinence, and need for parenteral nutrition.

The presence of interstitial lung disease was determined by using a clinical decision rule that was recently published [23]. This algorithm relies on physical examination (presence of typical "Velcro-like crackles" indicative of interstitial lung disease on lung auscultation), chest radiograph, and high-resolution computed tomography (HRCT). Interstitial lung disease is considered present if an HRCT lung study interpreted by an experienced radiologist shows interstitial lung disease or, in the case in which no HRCT was performed, if a chest radiograph is reported as showing either increased interstitial markings (not thought to be due to congestive heart failure) or fibrosis, and/or if a study physician reports the presence of typical Velcro-like crackles on physical examination.

Systolic pulmonary artery pressure (SPAP) is measured by using the Doppler flow measurement of the tricuspid regurgitant jet on echocardiography. Pulmonary hypertension (PH) was defined as an estimated systolic SPAP ≥ 45 mm Hg (an estimate that correlates strongly with right-heart catheter studies [24]).

Disease severity is assessed by using physician and patient global assessments of disease severity recorded on a numeric rating scale ranging from 0, representing no disease, to 10, representing most-severe disease. Disease severity also is measured by using a modified Medsger Scleroderma Disease Severity Scale [25,26]. The scale assesses disease severity in nine organ systems: general health, peripheral vascular, skin, joint/tendon, muscle, gastrointestinal tract, lungs, heart, and kidneys. Each organ is scored separately from 0 to 4, depending on whether no, mild, moderate, severe, or end-stage involvement is present. For the purposes of this study, the worst category was scored for each system, and results of any investigation not requested by the physician, and therefore missing, were considered "normal." Scoring methods for general, peripheral vascular, skin, joint/tendon, lung, and kidney systems were identical to those proposed in the scale. Some adaptations were made to the other organ systems. To assign a score for the skeletal muscle system, physicians were asked to rate patients' muscle strength in five different areas of the body (neck flexors, as well as upper and lower proximal extremities, right and left) by using the British Medical Research Council scale. A severity score was then assigned depending on the total number of 5s, 4s, 3s, 2s, 1s, and 0s for a given patient. The HAQ-DI was used to assess the patient's use of ambulation aids needed to assign the worst severity level for the skeletal muscle system (that is, level 4, end stage).

To score the gastrointestinal system, in addition to an abnormal esophagogram, abnormal esophageal manometry, or abnormal small-bowel series, patients reporting difficulty swallowing, acid taste in their mouth, choking at night, burning sensation, feeling of being full shortly after eating, or taking gastroprotective or promotility agents were also given a score of 1 for mild. In addition to malabsorption syndrome and episodes of pseudo-obstruction, patients with an abnormal hydrogen breath test were given a score of 3 for severe.

To score the heart system, electrocardiogram results, percentage left ventricular ejection fraction values, presence of conduction abnormalities, distended neck veins, and arrhythmia diagnosed by a physician were used.

### Statistical analysis

Descriptive statistics were used to summarize the baseline characteristics of the patients. χ^2 ^tests, Yates χ^2 ^tests, Fisher Exact tests, and Mann-Whitney *U *tests were used, as appropriate. *P *values < 0.05 were considered statistically significant. A sensitivity analysis was performed in the subset of patients who met the 1980 American College of Rheumatology preliminary classification criteria [27], of which 852 (89%) were found. All of the results were similar. Thus, the results of the whole cohort are presented here.

All statistical analyses were performed with SAS v.9.2 (SAS Institute, San Diego, CA, USA).

### Ethical considerations

Ethics committee approval for the CSRG data collection protocol was obtained at McGill University (Montreal, Ontario, Canada) and at all participating study sites. All subjects provided informed written consent to participate in the data-collection protocol.

## Results

This study included 963 SSc patients with complete serologic data on anti-Ro52/TRIM21 antibodies. Baseline characteristics of the study cohort are presented in Table 2. Mean age was 54.25 (± 12.04) years; 87% were female; 85% were White; mean disease duration (since the onset of the first non-Raynaud disease manifestation) was 11.04 (± 9.47) years; and 59% had limited, 37% had diffuse, and 3% had no skin involvement.

**Table 2:** Baseline characteristics of the study cohort, as a group and according to anti-Ro52/TRIM21 antibody status

	Whole group (*N* = 963)		Anti-Ro52/TRIM21 antibody positive (*n *= 194)		Anti-Ro52/TRIM21 antibody negative (*n *= 769)		Odds ratio (95% CI) and *P *values (Anti-Ro52/TRIM21 positive vs negative)	
			
	Mean or *n*	SD or %	Mean or *n*	SD or %	Mean or *n*	SD or %		
Age, years	55.42	12.04	57.16	11.45	54.98	12.16	1.02 (1.00, 1.03)	0.0481
Female, %	833	86.50%	175	90.21%	658	85.57%	NS	
White, %	816	84.74%	160	82.47%	656	85.31%	NS	
								
Disease duration, years	11.04	9.47	11.29	9.55	10.98	9.46	NS	
Disease subsets, %							NS	
Limited disease	563	59.26%	121	62.69%	442	58.39%		
Diffuse disease	356	37.47%	69	35.75%	287	37.91%		
Sine scleroderma	31	3.26%	3	1.55%	28	3.70%		
								
Modified Rodnan skin score (range, 0-51)	10.02	9.47	9.68	8.53	10.11	9.69	NS	
Swollen-joint count (range, 0-28)	0.91	3.34	1.07	4.26	0.87	3.07	NS	
Number of GI symptoms (range 0-14)	4.17	3.13	4.27	3.30	4.14	3.09	NS	
Inflammatory myositis, %	103	11.48%	21	11.54%	82	11.47%	NS	
Thrombosis, %	29	3.06%	7	3.65%	22	2.91%	NS	
Scleroderma renal crisis, %	42	4.44%	7	3.65%	35	4.64%	NS	
Interstitial lung disease, %	342	36.19%	85	44.27%	257	34.13%	1.53 (1.11, 2.12)	0.0091
Pulmonary hypertension,%	96	11.72%	26	15.38%	70	10.77%	NS	
								
Overlap with SLE, %	30	3.19%	8	4.23%	22	2.93%	NS	
Overlap with Sjögren, %	63	6.70%	18	9.52%	45	5.98%	NS	
Overlap with RA, %	30	3.19%	8	4.23%	22	2.93%	NS	
Overlap with MCTD, %	20	2.13%	7	3.70%	13	1.73%	NS	
Overlap with PM/DM, %	36	3.83%	12	6.35%	24	3.19%	2.06 (1.01, 4.19)	0.0431
Overlap syndrome, %	143	15.18%	41	21.58%	102	13.56%	1.75 (1.17, 2.63)	0.0059

CI, confidence interval; GI, gastrointestinal; MCTD, mixed connective tissue disease; NS, not significant; overlap syndrome, overlap with systemic lupus erythematosus, Sjögren syndrome, rheumatoid arthritis, mixed connective tissue disease, and/or polymyositis/dermatomyositis; PM/DM, polymyositis/dermatomyositis; RA, rheumatoid arthritis; SD, standard deviation; SLE, systemic lupus erythematosus.

Of the 963 SSc patients included in this study, 194 (20%) were positive for anti-Ro52/TRIM21 antibodies. This represents the second most common autoantibody in this cohort with anti-centromere, anti-RNA polymerase III, and anti-topoisomerase I antibodies present in approximately 35%, 19%, and 16% of the patients, respectively (Table 1). Anti-Ro52/TRIM21 antibody-positive patients were more likely to be older (57.16 versus 54.98 years; *P *= 0.0481), to have interstitial lung disease (44% versus 34%; *P *= 0.0091), and to have overlap syndrome (22% versus 14%; *P *= 0.0059) compared with anti-Ro52/TRIM21 antibody-negative patients (Table 2). In particular, anti-Ro52/TRIM21 antibody-positive patients were significantly more likely to have overlap with polymyositis/dermatomyositis (6% versus 3%; *P *= 0.0431) compared with anti-Ro52/TRIM21 antibody-negative patients. Also of note, two of 36 patients with polymyositis/dermatomyositis overlap had anti-Jo-1 antibodies (6%), and both of these had anti-Ro52/TRIM21 antibodies.

Disease severity measured by using the Medsger Disease Severity Scale was worse in two domains (range, 0 to 4): general (1.16 versus 0.82; *P *= 0.0061) and lung (1.56 versus 1.34; *P *= 0.0084) in anti-Ro52/TRIM21 antibody-positive compared with anti-Ro52/TRIM21 antibody-negative patients (Table 3).

**Table 3:** Association between Anti-Ro52/TRIM21 antibodies and disease severity (range, 0 to 4)

	Whole group (*N* = 963)		Anti-Ro52/TRIM21 antibody positive (*n *= 194)		Anti-Ro52/TRIM21 antibody negative (*n *= 769)		Odds ratio (95% CI) and *P *values (Anti-Ro52/TRIM21 positive vs negative)	
			
	Mean	SD	Mean	SD	Mean	SD		
General	0.89	1.22	1.16	1.41	0.82	1.16	1.23 (1.09, 1.39)	0.0061
Skin	1.25	0.68	1.23	0.60	1.25	0.69	NS	
Kidney	0.12	0.61	0.12	0.61	0.12	0.61	NS	
Peripheral vascular	1.54	1.25	1.58	1.26	1.53	1.24	NS	
Joint/tendon	0.73	1.19	0.73	1.22	0.73	1.18	NS	
Muscle	0.25	0.74	0.33	0.89	0.22	0.70	NS	
Gastrointestinal	1.95	0.82	2.01	0.78	1.93	0.82	NS	
Lung	1.38	1.11	1.56	1.08	1.34	1.12	1.19 (1.04, 1.37)	0.0084
Heart	0.50	1.00	0.60	1.12	0.48	0.97	NS	

Physician global assessments of severity (range, 0-10)	2.77	2.26	2.99	2.31	2.71	2.24	NS	
Patient global assessments of severity (range, 0-10)	3.64	2.61	3.66	2.67	3.63	2.60	NS	

CI, confidence interval; NS, not significant; SD, standard deviation.

Antibodies to Ro52/TRIM21 overlapped with many other autoantibodies (Table 4). However, the association between anti-Ro52/TRIM21 antibodies and other autoantibodies differed according to antibody. Indeed, whereas 16% of the overall cohort had anti-topoisomerase I antibodies, only 10% of anti-Ro52/TRIM21-positive patients had anti-topoisomerase I antibodies (*P *= 0.0129), and the titers of anti-Ro52/TRIM21 antibodies were among the lowest measured (mean titer, 763.28 U/ml) in this subset of patients. Conversely, whereas 6% of the overall cohort had anti-Ro60 antibodies, 21% of anti-Ro52/TRIM21 antibody-positive patients were anti-Ro60 positive (*P *< 0.0001), and the titers of anti-Ro52/TRIM21 antibodies were among the highest measured (mean titer, 5,530.66 U/ml). Similarly strong associations between anti-Ro52/TRIM21 antibodies and anti-Ro60 were noted between anti-Ro52/TRIM21 and anti-U1 RNP (*P *= 0.0066; mean titer, 2,099.45 U/ml), anti-SS-B/La (*P *< 0.0001; mean titer, 6,003.88 U/ml), and anti-Jo-1 (*P *= 0.0171; mean titer, 4,412.83 U/ml), and to a lesser degree between anti-Ro52/TRIM21 and anti-CENP-B (*P *= 0.0372; mean titer, 1,098.86 U/ml) and anti-Sm (*P *= 0.0361; mean titer, 1,482.09 U/ml) antibodies.

**Table 4:** Association between anti-Ro52/TRIM21 antibodies and other SSc-related autoantibodies

Antibody	Prevalence (*N* = 963)		Anti-Ro52 antibody positive (*n* = 194)		Anti-Ro52 antibody negative (*n* = 769)		*P *values	Mean anti-Ro52/TRIM21 titer (U/ml)
			
	*n*	*%*	*n*	*%*	*n*	%		
**Centromere**	334	34.76%	78	40.41%	256	33.33%	NS	1,098.64
**Topoisomerase I**	150	15.58%	19	9.79%	131	17.04%	0.0129	763.28
**RNApolIII**	144	18.58%	23	15.75%	121	19.24%	NS	1,164.47
**Pm/Scl**	53	6.84%	10	6.85%	43	6.84%	NS	1,381.13
**U1 RNP**	59	6.13%	20	10.31%	39	5.07%	0.0066	2,099.45
**CENP-B**	285	35.58%	64	42.95%	221	33.90%	0.0372	1,098.86
**CENP-A**	275	34.33%	61	40.94%	214	32.82%	NS	1,019.93
**Ro60**	53	5.50%	40	20.62%	13	1.69%	< 0.0001	5,530.66
**SS-B/La**	26	2.70%	18	9.28%	8	1.04%	< 0.0001	6,003.88
**Jo-1**	6	0.62%	4	2.06%	2	0.26%	0.0171	4,412.83
**Sm**	39	4.05%	13	6.70%	26	3.38%	0.0361	1,482.09
**Ribosomal P**	14	1.45%	3	1.55%	11	1.43%	NS	2,088.14
**Chromatin**	44	4.57%	10	5.15%	34	4.42%	NS	1,162.30

NS, not significant.

Finally, anti-Ro52/TRIM21 antibody titers were significantly higher in patients with compared to patients without overlap syndrome (mean titer, 1,330.40 versus 1,105.05 U/ml, respectively; *P *= 0.0026). A strong trend was noted toward higher titers in patients with compared to those without interstitial lung disease (mean titer, 1,542.65 versus 938.11 U/ml, respectively; *P *= 0.06).

## Discussion

In this large, multicenter cohort study, we found that anti-Ro52/TRIM21 antibodies were present in 20% of 963 patients, making them the second most common autoantibodies in this SSc cohort, and they overlapped with all of the major SSc-related antibodies. In addition, in this cohort of SSc patients, we found strong suggestions of an association between anti-Ro52/TRIM21 antibodies and interstitial lung disease and overlap syndrome.

Several studies have demonstrated that anti-Ro52/TRIM21 antibodies are present in several systemic autoimmune rheumatic diseases [11,28-31]. In particular, anti-Ro52 antibodies are frequently found in the inflammatory myositides, often in the presence of anti-Jo1 and interstitial lung disease [32-34].

The largest study to date of anti-Ro52 in SSc (measured using an ELISA), a recent British report of 1,010 SSc patients, found an overall frequency of anti-Ro52/TRIM21 antibodies of 27% [35]. We have shown that the ALBIA anti-Ro52/TRIM21 antibody has high concordance (> 95%) with an anti-Ro52/TRIM21 ELISA (unpublished data), suggesting that the higher prevalence in the British study is not related to interlaboratory assay variation. Although the majority of our patients were White (85%), the British study did not report on the ethnic profile or disease duration of their patients. The British study also showed that anti-Ro52/TRIM21 antibodies overlapped with all of the major SSc-related antibodies, including anti-centromere, anti-topoisomerase I, and anti-RNA polymerase III antibodies (in their study, at frequencies of 28%, 19%, and 25%, respectively). In addition, they confirmed that anti-Ro52/TRIM21 antibody titers were particularly elevated in patients with anti-Ro60 and anti-aminoacyl-tRNA synthetase antibodies (of whom four were anti-Jo1 positive). Although the authors did not present actual clinical data, they nevertheless concluded that Ro52/TRIM21 antibodies appeared to be general serum markers with limited linkage to distinct clinical manifestations of SSc.

In another recent study by Ghillani *et al. *[36], 155 patients positive for anti-Ro52/TRIM21 antibodies but negative for anti-Ro60 antibodies were analyzed. The authors found that anti-Ro52/TRIM21 antibodies were commonly found in the presence of other autoantibodies (in particular, Sm, chromatin, Jo-1, and CENP-B) and that 73% of patients had an autoimmune disease (in particular, polymyositis/dermatomyositis, SjS, SSc, and autoimmune hepatitis). Interestingly, the authors reported that the prevalence of pulmonary disease was particularly high among anti-Ro52/TRIM21 antibody-positive patients (22% of patients), including seven of 11 patients with SSc.

A recent article from the German Network for Systemic Sclerosis specifically examined the clinical correlates of a number of autoantibodies in SSc, including Ro52 [37]. They found that the prevalence anti-Ro52 antibody in their cohort (*N *= 863) was 21.7% and that 25% of patients with overlap syndrome were positive for anti-Ro52 antibody. Although they did not find any statistically significant association between anti-Ro52 antibodies and specific clinical features, it is interesting to note that their point estimate for an association with pulmonary fibrosis was 1.3. They also reported a statistically significant twofold increase in the rate of pulmonary fibrosis among patients who had anti-Ro60 antibodies (odds ratio (OR), 2.20; 95% confidence interval (CI), 1.29 to 3.75; *P *= 0.004). In our study, the rate of interstitial lung disease among anti-Ro52/TRIM21 antibody-positive patients was significantly increased by 50% compared with anti-Ro52/TRIM21 antibody-negative patients (OR, 1.53; 95% CI, 1.11 to 2.12; *P *= 0.0091). In addition, the association between anti-Ro60 antibody and interstitial lung disease was increased almost threefold (OR, 2.86; 95% CI, 1.62 to 5.04; *P *= 0.0002; data not shown).

Thus, the findings of our study are generally consistent with the current literature. In addition to confirming the serologic associations between anti-Ro52/TRIM21 antibodies and other autoantibodies, we found important associations between anti-Ro52/TRIM21 antibodies, interstitial lung disease, and overlap syndrome.

Overlap syndrome is highly prevalent among patients with systemic autoimmune rheumatic diseases and is thought to suggest that these diseases share similar immunogenetic mechanisms [38-40]. The concept of "overlap" syndrome in SSc, however, remains elusive, with some of the common manifestations of the disease, including sicca and myositis, being possibly related to SSc itself or to a separate autoimmune disease. We believe that serologic associations could help to shape a better definition of overlap in SSc, and that anti-Ro52/TRIM21 antibodies have the potential to be good biomarkers of overlap in this disease.

The Ro52 gene has been mapped to the end of the short arm of human chromosome 11 [29]. Evidence suggests that this chromosome segment may harbor genes important in the development and progression of solid tumors. Given this, we reviewed our data and found that 12.04% of anti-Ro52/TRIM21 antibody-positive patients compared with 7.24% of anti-Ro52/TRIM21 antibody-negative patients had a physician-reported history of malignancy (*P *= 0.03). These data have yet to be validated through chart review and remain preliminary.

Our understanding of how a number of SSc-related autoantibodies are generated (for example, through apoptosis, microbody release, molecular mimicry, or other mechanisms) and of their role in the pathogenesis of disease has increased remarkably over the past two decades (reviewed in [41,42]). The recently reported mechanism of intracellular immunity mediated by Ro52/TRIM21 in a cellular model of adenovirus infection has opened new perspectives for studying the effects of autoantibodies once they are inside cells [10]. The association between Ro52/TRIM21 and other proteins such as TRIB2, implicated in driving tumorigenesis [43], with Fas-associated death domain (FADD) and negatively regulating the IFN-α pathway in response to viral infection [44] and its interaction with virus-like particles and high-affinity IgG receptors affecting the intracellular fate of viruses, including degradation by the proteasomes [10,45,46] may provide important insights into the generation of the autoantibody response and its perpetuation in SSc. Further, the association of anti-Ro52/TRIM21 antibodies with cytokine responses such as IFN-α [44] and Toll-like receptor-mediated NF-κB activation [47] provides some insight into its regulation of cytokine responses. Thus, despite clearer understanding of the physiological function of Ro52/TRIM21 *in vitro*, it is still unclear how anti-Ro52/TRIM21 antibodies arise, particularly *in vivo*, and whether they have any pathologic significance in SSc or other systemic autoimmune rheumatic diseases. Of note, though, is the fact that anti-Ro52/TRIM21 antibodies are seen across a number of autoimmune diseases with significant prevalence (ranging from 1% to 63%) but are only rarely seen in normal controls; this suggests that they are markers of autoimmune dysfunction [29]. Further studies will be needed to shed greater insight into their precise role in autoimmune diseases.

## Conclusions

We believe that this study makes a significant contribution to the literature on the clinical significance of anti-Ro52/TRIM21 antibodies. Although consistent data show that SSc-specific antibodies are generally mutually exclusive, it is important to appreciate that a multiplicity of other nonspecific markers of humoral immune response exist in SSc, with Ro52/TRIM21 being one of the most common of these. In addition, Ro52/TRIM21 appears to have distinct clinical associations in SSc, in particular with interstitial lung disease and overlap syndrome. These data suggest that Ro52/TRIM21 may be of both diagnostic and prognostic importance in SSc.

## Abbreviations

ALBIA: addressable laser bead immunoassay; CENP: centromere protein; CI: confidence interval; CSRG: Canadian Scleroderma Research Group; ELISA: enzyme-linked immunosorbent assay; FADD: Fas-associated death domain; GI: gastrointestinal; HAQ-DI: Health Assessment Questionnaire-Disability Index; HRCT: high-resolution computed tomography; IgG: immunoglobulin G; IIF: indirect immunofluorescence; IFN: interferon; kDa: kiloDalton; MCTD: mixed connective tissue disease; NS: not significant; OR: odds ratio; PH: pulmonary hypertension; PM/DM: polymyositis/dermatomyositis; PM/Scl: polymyositis/scleroderma (exosome) autoantigen; RA: rheumatoid arthritis; RNA: ribonucleic acid; scRNP: small cytoplasmic ribonucleoprotein; SD: standard deviation; SjS: Sjögren syndrome; SLE: systemic lupus erythematosus; Sm: Smith autoantigen; SPAP: systolic pulmonary artery pressure; SS-A: Sjögren syndrome antigen A or Ro; SS-B: Sjögren syndrome antigen B or La; SSc: systemic sclerosis or scleroderma; TRIM: tripartite motif.

## Competing interests

The authors declare that they have no competing interests.

## Authors' contributions

MH participated in the design of the study, the data collection, and the statistical analysis, and prepared the manuscript. JP participated in the design of the study, the data collection, the statistical analysis, and the interpretation of the data. MM participated in the design of the study and the interpretation of the data. ST and RS performed the statistical analysis. MB and MF participated in the design of the study, the data collection, and the interpretation of the data. All co-authors read and approved the final manuscript. The Investigators of the Canadian Scleroderma Research Group participated in the acquisition of the data and read and approved the final manuscript.

## References

[B1] Steen VD Autoantibodies in systemic sclerosis Semin Arthritis Rheum 2005 35 35 42 10.1016/j.semarthrit.2005.03.005 16084222

[B2] Mahler M Raijmakers R Novel aspects of autoantibodies to the PM/Scl complex: clinical, genetic and diagnostic insights Autoimmun Rev 2007 6 432 437 10.1016/j.autrev.2007.01.013 17643929

[B3] Reveille JD Solomon DH Evidence-based guidelines for the use of immunologic tests: anticentromere, Scl-70, and nucleolar antibodies Arthritis Rheum 2003 49 399 412 10.1002/art.11113 12794797

[B4] Walker JG Fritzler MJ Update on autoantibodies in systemic sclerosis Curr Opin Rheumatol 2007 19 580 591 10.1097/BOR.0b013e3282e7d8f9 17917539

[B5] Arnett FC Gourh P Shete S Ahn CW Honey RE Agarwal SK Tan FK McNearney T Fischbach M Fritzler MJ Mayes MD Reveille JD Major histocompatibility complex (MHC) class II alleles, haplotypes and epitopes which confer susceptibility or protection in systemic sclerosis: analyses in 1300 Caucasian, African-American and Hispanic cases and 1000 controls Ann Rheum Dis 2010 69 822 827 10.1136/ard.2009.111906 19596691 PMC2916702

[B6] Robitaille G Christin MS Clement I Senecal JL Raymond Y Nuclear autoantigen CENP-B transactivation of the epidermal growth factor receptor via chemokine receptor 3 in vascular smooth muscle cells Arthritis Rheum 2009 60 2805 2816 10.1002/art.24765 19714638

[B7] Henault J Robitaille G Senecal JL Raymond Y DNA topoisomerase I binding to fibroblasts induces monocyte adhesion and activation in the presence of anti-topoisomerase I autoantibodies from systemic sclerosis patients Arthritis Rheum 2006 54 963 973 10.1002/art.21646 16508979

[B8] Henault J Tremblay M Clement I Raymond Y Senecal JL Direct binding of anti-DNA topoisomerase I autoantibodies to the cell surface of fibroblasts in patients with systemic sclerosis Arthritis Rheum 2004 50 3265 3274 10.1002/art.20515 15476238

[B9] Baroni SS Santillo M Bevilacqua F Luchetti M Spadoni T Mancini M Fraticelli P Sambo P Funaro A Kazlauskas A Avvedimento EV Gabrielli A Stimulatory autoantibodies to the PDGF receptor in systemic sclerosis N Engl J Med 2006 354 2667 2676 10.1056/NEJMoa052955 16790699

[B10] Racanelli V Prete M Musaraj G Dammacco F Perosa F Autoantibodies to intracellular antigens: generation and pathogenetic role Autoimmun Rev 2011 10 503 508 10.1016/j.autrev.2011.03.001 21397735

[B11] Hervier B Rimbert M Colonna F Hamidou MA Audrain M Clinical significance of anti-Ro/SSA-52 kDa antibodies: a retrospective monocentric study Rheumatology (Oxford) 2009 48 964 967 10.1093/rheumatology/kep145 19531627

[B12] Thompson AE Pope JE Increased prevalence of scleroderma in southwestern Ontario: a cluster analysis J Rheumatol 2002 29 1867 1873 12233880

[B13] Bernatsky S Joseph L Pineau CA Belisle P Hudson M Clarke AE Scleroderma prevalence: demographic variations in a population-based sample Arthritis Rheum 2009 61 400 404 10.1002/art.24339 19248123

[B14] Chifflot H Fautrel B Sordet C Chatelus E Sibilia J Incidence and prevalence of systemic sclerosis: a systematic literature review Semin Arthritis Rheum 2008 37 223 235 10.1016/j.semarthrit.2007.05.003 17692364

[B15] Lonzetti LS Joyal F Raynauld JP Roussin A Goulet JR Rich E Choquette D Raymond Y Senecal JL Updating the American College of Rheumatology preliminary classification criteria for systemic sclerosis: addition of severe nailfold capillaroscopy abnormalities markedly increases the sensitivity for limited scleroderma Arthritis Rheum 2001 44 735 736 10.1002/1529-0131(200103)44:3<735::AID-ANR125>3.0.CO;2-F 11263791

[B16] Hudson M Taillefer S Steele R Dunne J Johnson SR Jones N Mathieu JP Baron M Improving the sensitivity of the American College of Rheumatology classification criteria for systemic sclerosis Clin Exp Rheumatol 2007 25 754 757 18078627

[B17] Santiago M Baron M Hudson M Burlingame RW Fritzler MJ Antibodies to RNA polymerase III in systemic sclerosis detected by ELISA J Rheumatol 2007 34 1528 1534 17610318

[B18] Mahler M Raijmakers R Dahnrich C Bluthner M Fritzler MJ Clinical evaluation of autoantibodies to a novel PM/Scl peptide antigen Arthritis Res Ther 2005 7 R704 713 10.1186/ar1729 15899056 PMC1174964

[B19] Mahler M You D Baron M Taillefer SS Hudson M Canadian Scleroderma Research Group (CSRG) Fritzler MJ Anti-centromere antibodies in a large cohort of systemic sclerosis patients: comparison between immunofluorescence, CENP-A and CENP-B ELISA Clin Chim Acta 2011 412 1937 1943 10.1016/j.cca.2011.06.041 21756890

[B20] Clements P Lachenbruch P Siebold J White B Weiner S Martin R Weinstein A Weisman M Mayes M Collier D Inter- and intraobserver variability of total skin thickness score (modified Rodnan TSS) in systemic sclerosis J Rheumatol 1995 22 1281 1285 7562759

[B21] LeRoy EC Black C Fleischmajer R Jablonska S Krieg T Medsger TA Jr Rowell N Wollheim F Scleroderma (systemic sclerosis): classification, subsets and pathogenesis J Rheumatol 1988 15 202 205 3361530

[B22] van Gestel A Haagsma C van Riel P Validation of rheumatoid arthritis improvement criteria that include simplified joint counts Arthritis Rheum 1998 41 1845 1850 10.1002/1529-0131(199810)41:10<1845::AID-ART17>3.0.CO;2-K 9778226

[B23] Steele R Hudson M Lo E Baron M Canadian Scleroderma Research Group (CSRG) A clinical decision rule to predict the presence of interstitial lung disease in systemic sclerosis Arthritis Care Res (Hoboken) 2011 in press 10.1002/acr.21583 22213733

[B24] Hsu VM Moreyra AE Wilson AC Shinnar M Shindler DM Wilson JE Desai A Seibold JR Assessment of pulmonary arterial hypertension in patients with systemic sclerosis: comparison of noninvasive tests with results of right-heart catheterization J Rheumatol 2008 35 458 465 18203320

[B25] Medsger TA Jr Silman AJ Steen VD Black CM Akesson A Bacon PA Harris CA Jablonska S Jayson MI Jimenez SA Krieg T Leroy EC Maddison PJ Russell ML Schachter RK Wollheim FA Zacharaie H A disease severity scale for systemic sclerosis: development and testing J Rheumatol 1999 26 2159 2167 10529133

[B26] Medsger TA Jr Bombardieri S Czirjak L Scorza R Della Rossa A Bencivelli W Assessment of disease severity and prognosis Clin Exp Rheumatol 2003 21 S42 S46 12889222

[B27] Subcommittee for Scleroderma Criteria of the American Rheumatism Association Diagnostic and Therapeutic Criteria Committee Preliminary criteria for the classification of systemic sclerosis (scleroderma) Arthritis Rheum 1980 23 581 590 10.1002/art.1780230510 7378088

[B28] Peene I Meheus L Veys EM De Keyser F Diagnostic associations in a large and consecutively identified population positive for anti-SSA and/or anti-SSB: the range of associated diseases differs according to the detailed serotype Ann Rheum Dis 2002 61 1090 1094 10.1136/ard.61.12.1090 12429541 PMC1753972

[B29] Defendenti C Atzeni F Spina MF Grosso S Cereda A Guercilena G Bollani S Saibene S Puttini PS Clinical and laboratory aspects of Ro/SSA-52 autoantibodies Autoimmun Rev 2011 10 150 154 10.1016/j.autrev.2010.09.005 20854935

[B30] Dugar M Cox S Limaye V Gordon TP Roberts-Thomson PJ Diagnostic utility of anti-Ro52 detection in systemic autoimmunity Postgrad Med J 2010 86 79 82 10.1136/pgmj.2009.089656 20145055

[B31] Schulte-Pelkum J Fritzler M Mahler M Latest update on the Ro/SS-A autoantibody system Autoimmun Rev 2009 8 632 637 10.1016/j.autrev.2009.02.010 19393201

[B32] Rutjes SA Vree Egberts WT Jongen P Van Den Hoogen F Pruijn GJ Van Venrooij WJ Anti-Ro52 antibodies frequently co-occur with anti-Jo-1 antibodies in sera from patients with idiopathic inflammatory myopathy Clin Exp Immunol 1997 109 32 40 10.1046/j.1365-2249.1997.4081308.x 9218821 PMC1904727

[B33] Brouwer R Hengstman GJ Vree Egberts W Ehrfeld H Bozic B Ghirardello A Grondal G Hietarinta M Isenberg D Kalden JR Lundberg I Moutsopoulos H Roux-Lombard P Vencovsky J Wikman A Seelig HP van Engelen BG van Venrooij WJ Autoantibody profiles in the sera of European patients with myositis Ann Rheum Dis 2001 60 116 123 10.1136/ard.60.2.116 11156543 PMC1753477

[B34] Marie I Hatron PY Dominique S Cherin P Mouthon L Menard JF Levesque H Jouen F Short-term and long-term outcome of anti-Jo1-positive patients with anti-Ro52 antibody Semin Arthritis Rheum in press 10.1016/j.semarthrit.2011.09.008 22078416

[B35] Parker JC Burlingame RW Bunn CC Prevalence of antibodies to Ro-52 in a serologically defined population of patients with systemic sclerosis J Autoimmune Dis 2009 6 2 10.1186/1740-2557-6-2 19267890 PMC2654555

[B36] Ghillani P Andre C Toly C Rouquette AM Bengoufa D Nicaise P Goulvestre C Gleizes A Dragon-Durey MA Alyanakian MA Chretien P Chollet-Martin S Musset L Weill B Johanet C Clinical significance of anti-Ro52 (TRIM21) antibodies non-associated with anti-SSA 60 kDa antibodies: results of a multicentric study Autoimmun Rev 2011 10 509 513 10.1016/j.autrev.2011.03.004 21447407

[B37] Brouwer R Egberts WV Hengstman GJD Rajimakers R van Engelen BGM Seelig HP Renz M Mierau R Genth E Pruijn GJM Van Venrooij JV Autoantibodies directed to novel components of the PM/Scl complex, the human exosome Arthritis Res 2002 4 134 138 10.1186/ar389 11879549 PMC83843

[B38] Anaya JM Corena R Castiblanco J Rojas-Villarraga A Shoenfeld Y The kaleidoscope of autoimmunity: multiple autoimmune syndromes and familial autoimmunity Expert Rev Clin Immunol 2007 3 623 635 10.1586/1744666X.3.4.623 20477166

[B39] Hudson M Rojas-Villarraga A Coral-Alvarado P Lopez-Guzman S Mantilla RD Chalem P Baron M Anaya JM Polyautoimmunity and familial autoimmunity in systemic sclerosis J Autoimmun 2008 31 156 159 10.1016/j.jaut.2008.05.002 18644698

[B40] Rojas-Villarraga A Toro CE Espinosa G Rodriguez-Velosa Y Duarte-Rey C Mantilla RD Iglesias-Gamarra A Cervera R Anaya JM Factors influencing polyautoimmunity in systemic lupus erythematosus Autoimmun Rev 2010 9 229 232 10.1016/j.autrev.2009.10.001 19819350

[B41] Mackay IR Leskovsek NV Rose NR Cell damage and autoimmunity: a critical appraisal J Autoimmunity 2008 30 5 11 10.1016/j.jaut.2007.11.009 18194728 PMC2231525

[B42] Beyer C Pisetsky DS The role of microparticles in the pathogenesis of rheumatic diseases Nature Rev 2010 6 21 29 10.1038/nrrheum.2009.229 19949432

[B43] Grandinetti KB Stevens TA Ha S Salamone RJ Walker JR Zhang J Agarwalla S Tenen DG Peters EC Reddy VA Overexpression of TRIB2 in human lung cancers contributes to tumorigenesis through downregulation of C/EBPalpha Oncogene 2011 30 3328 3335 10.1038/onc.2011.57 21399661 PMC3382061

[B44] Young JA Sermwittayawong D Kim HJ Nandu S An N Erdjument-Bromage H Tempst P Coscoy L Winoto A Fas-associated death domain (FADD) and the E3 ubiquitin-protein ligase TRIM21 interact to negatively regulate virus-induced interferon production J Biol Chem 2011 286 6521 6531 10.1074/jbc.M110.172288 21183682 PMC3057824

[B45] Ohmine S Sakuma R Sakuma T Thatava T Takeuchi H Ikeda Y The antiviral spectra of TRIM5alpha orthologues and human TRIM family proteins against lentiviral production PLoS One 2011 6 e16121 10.1371/journal.pone.0016121 21264255 PMC3021539

[B46] Mallery DL McEwan WA Bidgood SR Towers GJ Johnson CM James LC Antibodies mediate intracellular immunity through tripartite motif-containing 21 (TRIM21) Proc Natl Acad Sci USA 2010 107 19985 19990 10.1073/pnas.1014074107 21045130 PMC2993423

[B47] Yoshimi R Chang TH Wang H Atsumi T Morse HC Ozato K Gene disruption study reveals a nonredundant role for TRIM21/Ro52 in NF-kappaB-dependent cytokine expression in fibroblasts J Immunol 2009 182 7527 7538 10.4049/jimmunol.0804121 19494276 PMC2803686

